# Molecular Mechanisms of the Gut–Liver–Placenta Axis in Intrahepatic Cholestasis of Pregnancy: Microbiota-Associated Pathogenesis and Next-Generation Therapeutic Strategies

**DOI:** 10.3390/ijms27198481

**Published:** 2026-09-23

**Authors:** Ningwei Liu, Shuairan Zhang, Yiling Li

**Affiliations:** Department of Gastroenterology, The First Affiliated Hospital of China Medical University, 155 Nanjing North Street, Shenyang 110001, China; lnw0110@126.com (N.L.); zhangshuairan@cmu.edu.cn (S.Z.)

**Keywords:** intrahepatic cholestasis of pregnancy, gut microbiota, bile acids, gut–liver–placenta axis, IBAT inhibitors

## Abstract

Intrahepatic cholestasis of pregnancy (ICP) is a pregnancy-specific liver disorder characterized by elevated maternal bile acid concentrations and an increased risk of adverse perinatal outcomes. Genetic susceptibility, hormonal changes, and impaired bile acid transport contribute to its development but do not fully explain the variability in disease severity and fetal outcomes. Increasing evidence suggests that gut microbiota dysbiosis and altered bile acid metabolism are associated with ICP and may participate in disease development through the gut–liver–placenta axis. In this review, we integrate evidence on bile acid–microbiota interactions during pregnancy, ICP-associated microbial and metabolic alterations, and their possible links to placental and fetal outcomes. Human studies primarily support associations among microbial alterations, bile acid profiles, and disease severity, whereas the microbiota-mediated effects on placental function and fetal injury proposed to date are supported largely by experimental models. We further discuss potential links among gut microbial dysfunction, placental oxidative stress, fetal cardiac injury, neurodevelopmental risk, offspring metabolic susceptibility, and iatrogenic prematurity. Although ursodeoxycholic acid (UDCA) improves maternal pruritus and selected biochemical indices, its effect on major perinatal outcomes remains uncertain. Ileal bile acid transporter (IBAT) inhibitors and microbiota-targeted interventions remain investigational, with insufficient pregnancy-specific evidence regarding their efficacy and maternal–fetal safety. Future studies should clarify the associated causal mechanisms and integrate metagenomic, metabolomic, and placental data to identify clinically relevant biomarkers and therapeutic targets.

## 1. Introduction

Intrahepatic cholestasis of pregnancy (ICP) is one of the most common pregnancy-specific liver disorders. It typically develops during the second or third trimester and is characterized by maternal generalized pruritus without a primary skin rash and elevated serum bile acid concentrations [1]. Maternal symptoms usually resolve after delivery [2]; however, ICP is associated with an increased risk of adverse perinatal outcomes, including preterm birth, meconium-stained amniotic fluid, fetal distress, and stillbirth [3].

The pathogenesis of ICP is multifactorial, with the major contributing factors including genetic susceptibility, pregnancy-related hormonal changes, and impaired bile acid transport; however, the precise interplay among these factors remains incompletely understood [2]. The conventional model attributes adverse pregnancy outcomes to the transplacental passage of high concentrations of maternal bile acids and their direct toxicity to placental trophoblasts [4]. Although such toxicity has been demonstrated in vitro, its relationship with clinical outcomes remains largely associative; while this model accounts for part of the clinical phenotype, it does not fully explain the heterogeneity in maternal manifestations and fetal outcomes observed in ICP [5,6].

Emerging evidence has separately implicated gut microbiota alterations, genetic susceptibility, and hormonal changes in ICP [2,7,8]. In addition to their hepatocellular toxicity, bile acids are major ecological determinants of the intestinal microbial community, and changes in their concentration and composition impose selective pressure on bile-sensitive taxa [7,9]. These changes may be accompanied by altered microbial metabolites and inflammatory signals, which have been proposed to influence the maternal–fetal interface through the gut–liver–placenta axis. At the placental level, experimental evidence from a murine model suggests that depletion of specific metabolites may impair the Nuclear factor erythroid 2-related factor 2.

(Nrf2)/Kelch-like ECH-associated protein 1 (Keap1) antioxidant defense system, thereby promoting trophoblast oxidative injury and fetal growth restriction [7]. At the fetal level, toxic bile acids [10] and inflammatory mediators [11] that cross the placental barrier may affect tissue development [10,11]. In the long-term, an adverse intrauterine metabolic environment may alter the offspring’s susceptibility to subsequent metabolic stress, as observed in an animal model [12].

Several recent reviews have discussed pathogenesis [13], bile acid metabolism [14], gut microbiota [14], or clinical management in the context of ICP [15]. However, these topics have largely been considered separately, and integrated perspectives regarding how intestinal microbial alterations interact with bile acid metabolism and the maternal–fetal interface remain limited. In this context, the gut–liver–placenta axis may help to explain the links between bile acid metabolism and changes in the intestinal microbiota in ICP. We discuss this relationship with particular attention to microbial metabolites and gut-derived inflammatory signals that could affect placental oxidative stress, inflammation, and fetal injury. Possible longer-term effects on offspring health are also considered, together with the potential and limitations of current therapies targeting the microbiota or bile acids.

The available evidence is still limited and comes from different types of studies. To date, most evidence linking gut microbial changes and bile acid profiles to ICP has come from observational studies in humans, whereas the proposed effects of microbial metabolites on placental function and fetal health have been investigated mainly in animal models. These findings suggest possible mechanisms, but whether they also occur in human ICP remains unclear.

This review summarizes recent advances in knowledge on the gut–liver–placenta axis, with particular emphasis on the interplay among the gut microbiota, bile acid metabolism, and host signaling pathways in ICP. We also discuss emerging therapeutic approaches, including inhibitors of the ileal bile acid transporter (IBAT) and microbiota-targeted interventions, and critically assess their potential relevance to the clinical management of ICP.

## 2. Physiological Basis of Bile Acid Enterohepatic Circulation and Gut Microbiota Remodeling During Pregnancy

### 2.1. Enterohepatic Circulation of Bile Acids and Nuclear Receptor Signaling

The enterohepatic circulation of bile acids constitutes a bidirectional interface between the gut microbiota and hepatic metabolism [16,17]. In humans, bile acid pool homeostasis is maintained through the coordinated actions of microbial biotransformation, ileal reabsorption, and nuclear receptor-mediated feedback regulation [13,16,17].

Primary bile acids—mainly cholic acid and chenodeoxycholic acid—are synthesized from cholesterol in hepatocytes, primarily through the classical pathway, in which cholesterol 7α-hydroxylase (*CYP7A1*) is the rate-limiting enzyme [18,19]. Newly synthesized primary bile acids are conjugated with glycine or taurine, which increases their aqueous solubility and reduces their cytotoxicity, and are subsequently secreted into bile canaliculi through the bile salt export pump, stored in the gallbladder, and released into the duodenum after food intake [20,21,22]. Upon reaching the terminal ileum and colon, conjugated bile acids are exposed to dense microbial communities and undergo extensive bacterial biotransformation [20]. Bacteria expressing bile salt hydrolase (BSH), including members of the phyla Bacteroidota and Bacillota, deconjugate bile salts and release unconjugated primary bile acids [23,24]. Bacterial species with 7α-dehydroxylating activity, particularly selected members of *Clostridium*, subsequently convert primary bile acids into secondary bile acids, such as deoxycholic acid and lithocholic acid [25].

Approximately 95% of intestinal bile acids are actively reabsorbed in the terminal ileum by the apical sodium-dependent bile acid transporter (ASBT)/solute carrier family 10 member 2 (SLC10A2), enter the portal circulation, and are taken up again by hepatocytes through the sodium taurocholate cotransporting polypeptide (NTCP) [26,27], thereby completing the enterohepatic cycle. Bile acids also act as potent signaling molecules by activating the nuclear receptor farnesoid X receptor (FXR) and the Takeda G protein-coupled receptor 5 (TGR5), also known as G protein-coupled bile acid receptor 1 (GPBAR1) [28]. Intestinal FXR activation induces the secretion of fibroblast growth factor 19 (FGF19). After reaching the liver through the portal circulation, FGF19 binds to the Fibroblast growth factor receptor 4 (FGFR4)/β-Klotho receptor complex and suppresses *CYP7A1* expression, thereby limiting de novo bile acid synthesis [29,30].

### 2.2. Physiological Remodeling of the Gut Microbiota During Normal Pregnancy

Pregnancy is accompanied by substantial endocrine and metabolic remodeling. As gestation progresses from the first to the second and third trimesters, the composition of the maternal gut microbiota also changes [31,32]. Previous studies have reported increased relative abundances of Proteobacteria and Actinobacteria and reduced phylogenetic diversity during late pregnancy [31,32]. In parallel, elevated progesterone levels may reduce gastrointestinal motility and prolong intestinal transit, as observed in pregnant mice [33]. However, evidence for these effects during pregnancy remains limited.

The relationship between bile acids and the gut microbiota is bidirectional and involves microbial biotransformation, receptor signaling, and feedback regulation. Under physiological conditions, this network maintains both bile acid pool composition and intestinal ecological stability. The major components of this physiological network—including enterohepatic bile acid circulation, microbial transformation, and bile acid receptor signaling—are summarized in Figure 1. The following section examines how genetic susceptibility and pregnancy-related hormonal changes disrupt bile acid homeostasis in ICP.

## 3. Pathological Initiation of ICP: Genetic Susceptibility and Pregnancy-Related Hormonal Load

### 3.1. Genetic Susceptibility to ICP

Genetic susceptibility is an important contributor to ICP, with ATP-binding cassette subfamily B member 4 (*ABCB4*) and ATP-binding cassette subfamily B member 11 (*ABCB11*) being among the most extensively studied genes [34,35]. *ABCB4* encodes multidrug resistance protein 3 (MDR3), a phospholipid transporter located on the canalicular membrane, whereas *ABCB11* encodes the bile salt export pump (BSEP); both proteins contribute to bile formation and bile acid excretion [36,37]. Pathogenic variants in these genes reduce the hepatocellular transport of phospholipids and bile acids [38] and are therefore more frequently identified in patients with severe or early-onset ICP [34,38]. However, genetic factors alone are generally insufficient to cause the disease. Since most patients develop symptoms only during pregnancy and gradually recover after delivery, pregnancy-specific factors—particularly elevated sulfated progesterone metabolites—likely play a critical role in unmasking the cholestatic phenotype in genetically susceptible individuals [37,39]. Pregnancy-related increases in estrogens and progesterone metabolites may place additional functional demands on hepatic bile acid transport and excretion, in part by interfering with key hepatobiliary transporters and nuclear receptors such as BSEP, NTCP, MDR3, and FXR [40,41]. Under physiological conditions, compensatory mechanisms maintain bile acid homeostasis. Experimental models have suggested that impaired hepatobiliary transport can increase susceptibility to estrogen-induced disturbances in bile acid homeostasis, thereby aggravating defects in bile acid excretion [40]. Genetic factors therefore confer susceptibility to ICP, whereas pregnancy-related hormonal changes act as important triggers of disease onset.

### 3.2. Hormone-Driven Impairment of Bile Acid Excretion and Bile Acid Overload

Elevated concentrations of estrogens and progesterone metabolites during the second and third trimesters can impair hepatocellular bile acid transport. Estrogens can suppress BSEP expression, whereas certain sulfated progesterone metabolites antagonize FXR-mediated transcriptional activation of BSEP, further impairing bile acid export [39,41,42]. In the setting of genetic susceptibility and increased pregnancy-related hormonal load, canalicular bile acid secretion can become restricted, leading to the progressive accumulation of bile acids in the liver and maternal circulation and, ultimately, to the characteristic disruption of bile acid homeostasis in ICP [39,42,43].

Altered bile acid homeostasis is associated with changes in the intestinal bile acid pool and its bacterial modification, with potential consequences for the structure of the gut microbiota and metabolic activity; however, the causal relationship is likely bidirectional and remains incompletely defined in ICP [44]. Clinical studies have identified alterations in both the gut microbiota and fecal metabolite profiles of patients with ICP, with more pronounced changes in those with severe disease [45]. Animal studies have further suggested that gut microbial dysbiosis can alter enterohepatic feedback regulation by modifying bile acid deconjugation and biotransformation, thereby contributing to disturbances in bile acid metabolism [46]. Thus, the bile acid abnormalities and gut microbial dysbiosis observed in ICP are more likely to interact and evolve together than to represent a simple unidirectional causal relationship [47].

## 4. Gut Microbiota Alterations Associated with Bile Acid Disturbances in ICP

### 4.1. Altered Gut Microbial Composition in ICP

Recent cohort studies using 16S rRNA gene sequencing and high-resolution metagenomics have characterized the gut microbiota of patients with ICP at both the taxonomic and functional levels.

At the phylum level, studies have reported a lower relative abundance of Firmicutes (Bacillota) in ICP, particularly in severe disease, with several short-chain fatty acid (SCFA)-producing genera within this phylum—including *Faecalibacterium*, *Blautia*, and *Eubacterium*—also showing reduced abundance. However, the severity-specific depletion of these genera is best documented for *Eubacterium* subgroups [45,48]. The depletion of these taxa may be related to the altered SCFA profile observed in ICP, although the available studies do not establish a direct causal relationship between their depletion and disease severity. In contrast, Proteobacteria have been reported to be more abundant in patients with severe ICP [45]. Higher Proteobacteria abundance has also been associated with higher maternal serum bilirubin levels [49]. These observations indicate an association between gut microbial composition and the biochemical severity of ICP, but do not establish whether microbial changes precede or result from cholestasis.

Reduced abundance of *Megamonas* has also been reported in patients with ICP [50]. *Megamonas* species—as exemplified by *M. funiformis*—are known to produce SCFAs, particularly propionate and acetate [50,51]. However, whether the lower abundance of *Megamonas* is associated with the metabolic changes reported in ICP remains unclear. It is also uncertain whether this reduction reflects a response to the altered bile acid environment or contributes independently to the pathogenesis of ICP.

Several taxa, including *Flavonifractor*, *Atopobium*, *Turicibacter*, *Parabacteroides*, and *Escherichia–Shigella*, were found to be more abundant in ICP; enrichment of *Escherichia–Shigella* was particularly evident in patients with severe disease and was accompanied by distinct fecal metabolic profiles [45,50]. 16S rRNA-based analyses have identified differences in the predicted functional profiles of the gut microbiota in the context of ICP, including alterations in pathways related to unsaturated fatty acid biosynthesis and propanoate metabolism [50]. Other 16S rRNA gene amplicon studies have also identified broad differences in predicted microbial metabolic pathways between ICP and healthy pregnancy [52]. Shotgun metagenomic analyses have further identified functional differences in the ICP microbiome, particularly in pathways involved in bile acid metabolism [46]. These findings suggest that ICP is associated with differences in both microbial composition and functional profiles. However, most human studies to date have been observational, and these data are not sufficient to determine whether the observed functional changes contribute to ICP or arise as a consequence of the altered physiological environment.

### 4.2. Functional and Metabolic Alterations of the Gut Microbiota in ICP

Changes in the gut microbiota in ICP are also associated with alterations in microbial metabolism [50,52]. Functional profiling based on 16S sequencing and metagenomic analyses has identified differences in microbial pathways related to bile acid and other metabolic processes, and metabolomic analyses have revealed alterations in fecal and circulating metabolites, particularly in the context of severe ICP. However, whether these changes in bile-acid-related microbial functions measurably affect host bile acid homeostasis remains incompletely defined in humans [45,46,48,50,52]. Experimental studies have suggested that the gut microbiota can modify the bile acid composition and intestinal FXR signaling [46,53]. Consistent with this possibility, the transfer of microbiota from patients with ICP induced cholestatic features in mice [46]. These experimental findings provide mechanistic support for a role of the gut microbiota in bile acid regulation, but whether similar changes contribute directly to ICP development in humans remains uncertain [46,53].

Fecal metabolomic analyses have also identified differences according to disease severity [45]. In a metabolomic analysis of fecal samples, severe ICP was associated with reduced butyrate and hypoxanthine and elevated succinate and formate relative to mild ICP or healthy pregnancies [45]. Model-based Integration of Metabolite Observations and Species Abundances (MIMOSA) analysis of the same cohort revealed associations between hypoxanthine levels and several bacterial taxa, including *Ruminococcus gnavus* and members of the Lachnospiraceae family [45]. In severe ICP, reduced fecal hypoxanthine was associated with specific microbial taxa predicted to contribute to hypoxanthine metabolism, suggesting that altered microbial metabolism may contribute to this change; however, both the microbial and metabolic alterations may also reflect the broader metabolic disturbance occurring under the conditions of severe ICP [45,54]. In addition to hypoxanthine, severe ICP was also associated with altered levels of other metabolites, including butyrate and succinate. Severe ICP was associated with reduced fecal butyrate and increased succinate levels [45]. Altered circulating SCFA profiles, including reduced butyrate, have also been reported in an independent ICP cohort [55]; however, these alterations do not establish a pathogenic role alone.

Circulating metabolite profiles provide some additional evidence of altered SCFA metabolism in ICP. Lower butyrate levels have been observed in maternal serum, with a similar declining trend in umbilical cord blood [55]. Other metabolomic studies have also identified alterations in circulating metabolites, including changes in bile acids, lipids, amino acid derivatives, and other metabolic pathways in patients with ICP [56,57,58,59,60]. Although serum concentrations may not directly reflect changes in the intestinal lumen, these findings suggest that the metabolic alterations associated with ICP are not confined to the gut. Functional profiling of the gut microbiota in severe ICP has also identified the enrichment of pathways related to unsaturated fatty acid biosynthesis and propanoate metabolism [50]. These changes suggest that the metabolic alterations associated with ICP extend beyond bile acid and purine metabolism [59,60].

Overall, current human studies support associations among gut microbial alterations, microbial metabolites, and disease severity in ICP [45,48,50,52]. However, most available studies are observational and the temporal relationship between microbial alterations and ICP remains difficult to establish. Recent longitudinal data from a large pregnancy cohort suggest that some gut microbial changes may precede the clinical diagnosis of ICP, although whether these alterations contribute to disease development or reflect early metabolic disturbances remains uncertain [61]. Experimental studies provide additional mechanistic clues, particularly regarding microbiota-mediated regulation of bile acid metabolism and FXR signaling [46,62,63], but their relevance to human pregnancy remains uncertain. The relationship between the gut microbiota and bile acid metabolism in ICP may involve bidirectional interactions, but a causal pathway has not yet been established.

## 5. Adverse Fetal Outcomes Associated with Dysregulation of the Gut–Liver–Placenta Axis

### 5.1. Perinatal Injury

ICP is associated with a range of perinatal complications, including preterm birth, meconium-stained amniotic fluid, fetal distress, and stillbirth [64,65,66]. The risk of these outcomes varies with the severity of maternal cholestasis. Serum total bile acid concentrations show a tight association with stillbirth [64,66]. In a large meta-analysis of individual participant data, the risk of stillbirth increased markedly when maternal total bile acid concentrations reached ≥100 μmol/L, whereas the risk at lower concentrations was substantially smaller [64]. However, the mechanisms underlying fetal injury remain incompletely understood.

The placenta is exposed to the altered bile acid environment in ICP, and changes in placental bile acid transporters, morphology, and oxidative stress have been reported [67,68,69]. Experimental studies have further indicated that elevated bile acids can affect trophoblast function and placental vascular tone [69,70,71]. Bile acids can also alter cardiomyocyte contraction and calcium handling, and clinical studies have reported changes in the fetal PR interval and cardiac function in ICP [72]. Although these findings support biologically plausible effects of excess bile acid exposure on the placenta and fetus, the extent to which these changes account for acute fetal deterioration remains uncertain.

In a mouse model of gestational cholestasis, gut microbiota alterations were associated with reduced valeric acid and erythritol concentrations, changes in placental Nrf2/Keap1-related antioxidant responses, oxidative stress, and fetal growth restriction [7], providing experimental evidence that intestinal microbial metabolism may be linked to placental responses during cholestasis.

Overall, clinical evidence linking maternal bile acid concentrations with adverse perinatal outcomes is more extensive than that available for microbiota-mediated mechanisms. Whether gut microbial alterations contribute to fetal injury in human ICP remains unclear. Current decisions regarding delivery are based primarily on maternal bile acid concentrations, gestational age, and other clinical factors, balancing fetal risk against the consequences of preterm birth [64,73].

### 5.2. Postnatal Injury and Long-Term Consequences

The consequences of ICP may extend beyond the perinatal period. A nationwide Swedish cohort of approximately two million children found that maternal ICP was associated with a higher risk of several neurodevelopmental conditions, particularly after early-onset ICP [74]. Altered fetal bile acid exposure and placental inflammatory or stress responses have been proposed, but these mechanisms have not been directly examined in children exposed to ICP [11].

Long-term metabolic consequences have been investigated mainly in experimental settings, where gestational cholestasis has been linked to persistent changes in bile acid metabolism [12], hepatic lipid handling, and gut microbial composition in offspring [12,63,75]. In experimental models, maternal hypercholanemia has been associated with altered offspring glucose and lipid metabolism, and these changes may become more apparent when offspring are subsequently exposed to a high-fat or Western-type diet [12,75,76]. Whether similar metabolic phenotypes occur in children born to women with ICP remains unclear.

Human studies have reported metabolic alterations in neonates exposed to maternal ICP [77], and maternal glucose/lipid changes have been associated with increased fetal growth [78]; however, evidence that these changes persist into later life is limited [77,78].

Preterm birth may also affect postnatal gut development in pregnancies complicated by ICP. Preterm infants commonly show delayed establishment of the gut microbiota, with gestational age, antibiotic exposure, parenteral nutrition, and enteral feeding all influencing microbial colonization [79,80,81]. Changes in bile acid metabolism are also observed during early microbial development in preterm infants, and reduced microbial bile salt hydrolase activity has been reported in preterm infants with cholestasis [82,83]. These findings provide a relevant background for considering the gut–liver axis after preterm delivery, but they have not been specifically demonstrated in infants delivered preterm because of ICP.

Current human evidence provides support for an association between maternal ICP, especially early-onset ICP, and later neurodevelopmental outcomes [74]. Similarly, the available evidence on neonatal gut–liver axis development comes mainly from studies of prematurity or neonatal cholestasis rather than ICP itself [79,80,81,82,83]. Taken together, the current evidence suggests links among bile acid dysregulation, gut microbial alterations, placental dysfunction, and offspring outcomes, although the strength of evidence differs across these pathways. Figure 2 summarizes this framework and distinguishes relatively well-supported associations from proposed or experimentally derived mechanisms.

## 6. Limitations of Current Therapy and Emerging Precision Interventions

### 6.1. Ursodeoxycholic Acid: Therapeutic Effects and Limitations

Ursodeoxycholic acid (UDCA) remains one of the most widely used pharmacological treatments for intrahepatic cholestasis of pregnancy (ICP) and is recommended for relief of maternal pruritus [84,85]. Its anticholestatic effects are generally attributed to a reduction in bile acid cytotoxicity, stimulation of hepatobiliary secretion, and protection against bile acid-induced cellular injury [86,87]. UDCA may also alter the intestinal bile acid pool and microbial bile acid metabolism [88], although whether these changes mediate clinical outcomes in ICP remains to be determined [47,88].

Clinical evidence supports the efficacy of UDCA in improving maternal pruritus and selected liver biochemical indices. Earlier randomized trials, observational studies and meta-analyses reported improvements in pruritus and liver biochemical indices [89,90,91,92]. The PITCHES trial subsequently showed that UDCA did not significantly reduce the composite outcome of perinatal death, preterm birth, or neonatal unit admission [93]. An individual participant data (IPD) meta-analysis also found no significant reduction in stillbirth overall, although some analyses of randomized trials suggested a possible reduction in the combined outcome of stillbirth and preterm birth [94]. These findings underscore the need to distinguish improvement in maternal symptoms and biochemical markers from prevention of major fetal complications.

Other pharmacological approaches, including S-adenosylmethionine and rifampicin, have been evaluated mainly in smaller trials, observational studies, or as adjunctive therapy in patients with an inadequate response to UDCA [95,96,97,98]. The available studies suggest possible symptomatic or biochemical benefit, but the evidence is not sufficient to establish a reduction in clinically important perinatal outcomes. Representative clinical efficacy and maternal–fetal safety data are summarized in Table 1.

Experimental and translational evidence indicates that UDCA can improve fetal and placental lipid profiles and may thereby modify the fetal–placental metabolic environment [75], but these observations do not alter its current clinical role. The most consistent benefit of UDCA is a small improvement in maternal pruritus; evidence for perinatal benefit is limited and inconsistent overall, although some guidelines and IPD analyses support a possible reduction in preterm birth and a composite of stillbirth and preterm birth in severe ICP. UDCA therefore does not replace serial bile acid monitoring, risk stratification, or appropriately timed delivery [84,85,93,94,95]. Current major guidelines are consistent in retaining UDCA principally for maternal symptoms while basing fetal risk management heavily on bile acid concentrations and gestational age.

### 6.2. IBAT Inhibitors: Interrupting the Enterohepatic Circulation of Bile Acids

Inhibition of the ileal bile acid transporter (IBAT) provides an alternative therapeutic approach by reducing active ileal bile acid reabsorption and increasing fecal bile acid excretion [99,100]. By interrupting enterohepatic recycling, IBAT inhibition can reduce the circulating bile acid burden. Clinical studies of odevixibat and maralixibat in progressive familial intrahepatic cholestasis (PFIC) and Alagille syndrome have demonstrated reductions in pruritus and, in a substantial proportion of patients, serum bile acid concentrations [101,102,103,104,105].

These results establish the pharmacological activity of IBAT inhibition in cholestatic disease, but they cannot be directly extrapolated to ICP. PFIC and Alagille syndrome are chronic, predominantly pediatric disorders, whereas ICP is a pregnancy-specific condition that requires separate evaluation of efficacy and safety [99,100,106].

The OHANA study provides preliminary evidence for IBAT inhibition in ICP. Volixibat was associated with an improvement in pruritus in three of four women and with marked reductions in serum bile acids, but only four women received the treatment [107]. Gastrointestinal adverse events were observed but, due to the small sample size, safety or effects on stillbirth, spontaneous preterm birth, fetal distress, or other major perinatal outcomes could not be established [107]. More recent reports describing the use of maralixibat during pregnancy provide additional clinical experience but remain limited to individual cases and thus cannot establish efficacy or maternal–fetal safety [108,109].

IBAT inhibitors therefore remain investigational in the context of ICP. Larger pregnancy-specific studies are needed to define dosing, tolerability, maternal pharmacokinetics, fetal exposure, and whether reductions in circulating bile acids translate into better perinatal outcomes. The available efficacy and safety evidence is summarized in Table 1.

### 6.3. Microbiota-Targeted Interventions

Changes in gut microbial composition and function provide a biological rationale for microbiota-targeted interventions in ICP [46]. Human studies have identified associations of ICP with altered microbial composition, bile acid metabolism, and microbial metabolites [45,47,49], while gut microbiota dysbiosis and altered microbial metabolites have been associated with placental dysfunction in experimental models [7]. However, most human evidence remains observational, and evidence for therapeutic effects is derived largely from animal models.

Importantly, evidence from randomized controlled trials demonstrating the efficacy of probiotics, postbiotics, or other microbiota-targeted interventions specifically in pregnant women with ICP is currently lacking [47]. In animal models of ICP, specific probiotics such as *Roseburia intestinalis* and *Lactobacillus paragasseri* LPG-9 have shown beneficial effects on bile acid metabolism; LPG-9 has also been shown to reduce placental inflammation [53,110].

Postbiotics may offer advantages in terms of stability and standardization [111]. According to the International Scientific Association for Probiotics and Prebiotics (ISAPP) consensus, postbiotics refer to preparations of inanimate microorganisms or their components, rather than purified microbial metabolites alone [112,113]. Both postbiotic preparations and microbiota-derived metabolites remain experimental in ICP, with limited information on effective exposure, placental effects, fetal development, and interactions with maternal bile acid metabolism [113].

Microbiota-targeted therapy should therefore remain an experimental approach in ICP. Future studies should prioritize pregnancy-specific safety assessment and adequately powered clinical trials before these interventions can be considered for routine clinical use.

**Table 1:** Current and emerging therapeutic strategies for intrahepatic cholestasis of pregnancy (ICP): mechanisms, clinical evidence, and major limitations.

Article	Study Design /Sample Size	Main Clinical Data	Safety Data
Chappell et al. [93]	RCT, *n* = 605	UDCA did not significantly reduce the composite outcome of perinatal death, preterm delivery, or neonatal unit admission for at least 4 h; UDCA 23.0% vs. placebo 26.7%; adjusted RR 0.85 (95% CI 0.62–1.15)	SAEs were 2 in the UDCA group and 6 in the placebo group, and none were considered treatment-related
Ovadia et al. [94]	Meta-analysis, 34 studies, 6974 women, 7135 fetuses	Overall stillbirth did not differ, aOR 1.04 (95% CI 0.35–3.07). In RCT-only analysis, the composite of stillbirth or preterm birth was reduced, aOR 0.60 (95% CI 0.39–0.91)	Not reported
Kondrackiene et al. [90]	RCT, *n* = 84	Pruritus improvement: UDCA 67% (28/42) vs. cholestyramine 19% (8/42)	No adverse effects were reported in the UDCA group; 12/42 (29%) in the cholestyramine group had nausea, vomiting, or diarrhoea
Glantz et al. [91]	RCT, *n* = 130	UDCA reduced only ALT and bilirubin, not bile acids or pruritus. In the severe ICP subgroup (bile acids ≥ 40 μmol/L), UDCA improved pruritus, bile acids, ALT, and bilirubin, but did not improve fetal complications	1 diarrhoea in the UDCA group; 1 nausea/dizziness/stomach pain in the dexamethasone group; 1 severe headache in the placebo group; 1 stillbirth occurred in the placebo group
Parizek et al. [92]	Retrospective multicentre cohort, *n* = 447	Pruritus improved in 86% and ALT/AST in 70%	Skin reactions 0.5%, mild diarrhoea 4.7%, and no treatment-attributable fetal/neonatal complications
Zhang et al. [98]	RCT, *n* = 120	UDCA was better than SAMe for reducing TBA; UDCA and combination were better than SAMe for AST and TB	No perinatal deaths and no adverse drug reactions
Roncaglia et al. [114]	RCT, *n* = 46	Both UDCA and SAMe improved pruritus, but UDCA was superior to SAMe in reducing serum bile acids, AST, ALT, and bilirubin	No adverse fetal or neonatal effects were noted with either therapy
Ribalta et al. [115]	RCT, *n* = 18	SAMe: no significant improvement in pruritus, bile acids, ALT, bilirubin, or alkaline phosphatase	No relevant adverse reactions were detected
Geenes et al. [97]	A case series, *n* = 27	54% had TBA reduction after adding rifampicin, 38% had ≥50% reduction	No obvious adverse effects were reported
Walker et al. [95]	Systematic review, 26 studies, 2007 women	UDCA may provide a small improvement in pruritus, but evidence regarding its effect on stillbirth and fetal distress remains uncertain	Most non-UDCA intervention studies had small sample sizes and a high risk of bias, and therefore cannot provide strong conclusions regarding maternal and fetal safety
Kumari et al. [96]	Prospective observational study, *n* = 108	In UDCA non/partial responders with ICP, 24/30 women reported relief of pruritus after addition of rifampicin	Earlier delivery and lower birth weight

Note: This table summarizes both established and investigational therapeutic approaches for ICP. UDCA remains the principal pharmacological treatment for maternal pruritus and selected biochemical abnormalities, although evidence for improvement in major perinatal outcomes remains inconsistent. IBAT inhibitors reduce ileal bile acid reabsorption and have demonstrated pharmacological activity in other cholestatic disorders, but direct evidence in ICP is currently limited and pregnancy-specific maternal–fetal safety has not been established. Microbiota-targeted approaches, including function-guided probiotics, next-generation probiotics, postbiotics, and microbiota-derived metabolites, remain experimental; available evidence is derived predominantly from mechanistic studies and animal models, with little or no intervention evidence in pregnant women with ICP. Representative clinical studies are included to illustrate the efficacy and safety data available for UDCA, S-adenosylmethionine, rifampicin, and related treatment strategies. Differences in study design, sample size, disease severity, treatment regimen, and outcome definitions should be considered when comparing results across studies. The absence of reported adverse events in individual studies should not be interpreted as definitive evidence of maternal–fetal safety. Abbreviations: ALT, alanine aminotransferase; AST, aspartate aminotransferase; aOR, adjusted odds ratio; BSH, bile salt hydrolase; CI, confidence interval; IBAT, ileal bile acid transporter; ICP, intrahepatic cholestasis of pregnancy; NGPs, next-generation probiotics; PFIC, progressive familial intrahepatic cholestasis; RCT, randomized controlled trial; RR, relative risk; SAE, serious adverse event; SAMe, S-adenosyl-L-methionine; SCFA, short-chain fatty acid; TBA, total bile acid; TB, total bilirubin; UDCA, ursodeoxycholic acid.

## 7. Multi-Omics Integration and Future Diagnostic and Therapeutic Directions

ICP is a complex pregnancy-associated disorder involving genetic background, bile acid metabolism, the gut microbiota, and placental function [13,54,84,116]. At present, clinical assessment relies primarily on serum total bile acid concentrations and liver biochemical tests. Although these measures are important for diagnosis and risk stratification, they do not fully capture changes in individual bile acid species, microbial function, or placental responses [117,118].

Metagenomics and metabolomics can characterize changes in microbial functions, bile acids, SCFAs, and other microbiota-associated metabolites [45,46,55]. Transcriptomic and single-cell approaches may further resolve molecular changes across placental cell types [119,120]. Maternal, intestinal, placental, and fetal data may help to clarify whether these changes are related and whether they occur in a consistent temporal sequence, although the current evidence remains insufficient and future longitudinal multi-omics studies are needed [55,61,116].

Bile acid metabolomics with clinical indicators, as well as machine learning models based on routine clinical and laboratory indicators, may improve early diagnosis and risk prediction of ICP [121,122,123]. However, the current evidence remains limited by small sample sizes, population heterogeneity, inconsistent sampling schedules, and insufficient external validation [116,121,122,124]. Moreover, multi-omics associations do not by themselves establish causality [61,125]. Future studies are needed to distinguish predictive biomarkers from factors that directly contribute to disease.

Similarly, microbial or metabolic targets identified through multi-omics studies should not be translated directly into clinical treatment. Their biological relevance and maternal–fetal safety need to be established before clinical evaluation, particularly for microbiota-targeted interventions.

## 8. Conclusions and Perspectives

ICP is a multifactorial pregnancy-associated cholestatic disorder in which genetic susceptibility, pregnancy-related hormonal changes, and impaired bile acid transport interact to disrupt bile acid homeostasis. Alterations in the gut microbiota and microbial metabolism have also been associated with ICP, although their contribution to disease initiation and progression remains incompletely defined. The gut–liver–placenta axis therefore provides a useful framework for integrating maternal bile acid dysregulation, microbial changes, placental dysfunction, and fetal outcomes, but should be viewed as a conceptual framework rather than an established causal pathway in human ICP.

The current clinical evidence for the association between maternal bile acid concentrations and adverse perinatal outcomes is strong, whereas microbiota-mediated effects on placental function and fetal injury are supported mainly by observational studies and experimental models. This difference in the strength of evidence is also relevant when considering treatment. UDCA remains the principal treatment for maternal symptoms and selected biochemical abnormalities, but its benefit for major perinatal outcomes remains uncertain. IBAT inhibitors and microbiota-targeted interventions remain investigational, and pregnancy-specific evidence regarding their efficacy and maternal–fetal safety remains limited.

Future studies should prioritize longitudinal pregnancy cohorts and integrate bile acid profiling, metagenomics, metabolomics, and placental molecular data with mechanistic experiments. Such approaches may help to distinguish biomarkers that predict disease severity from factors that directly contribute to pathogenesis. Clarifying the temporal and causal relationships within the gut–liver–placenta axis will be necessary before microbiota- or metabolite-based targets can be translated into clinically applicable interventions for ICP.

## Figures and Tables

**Figure 1:**
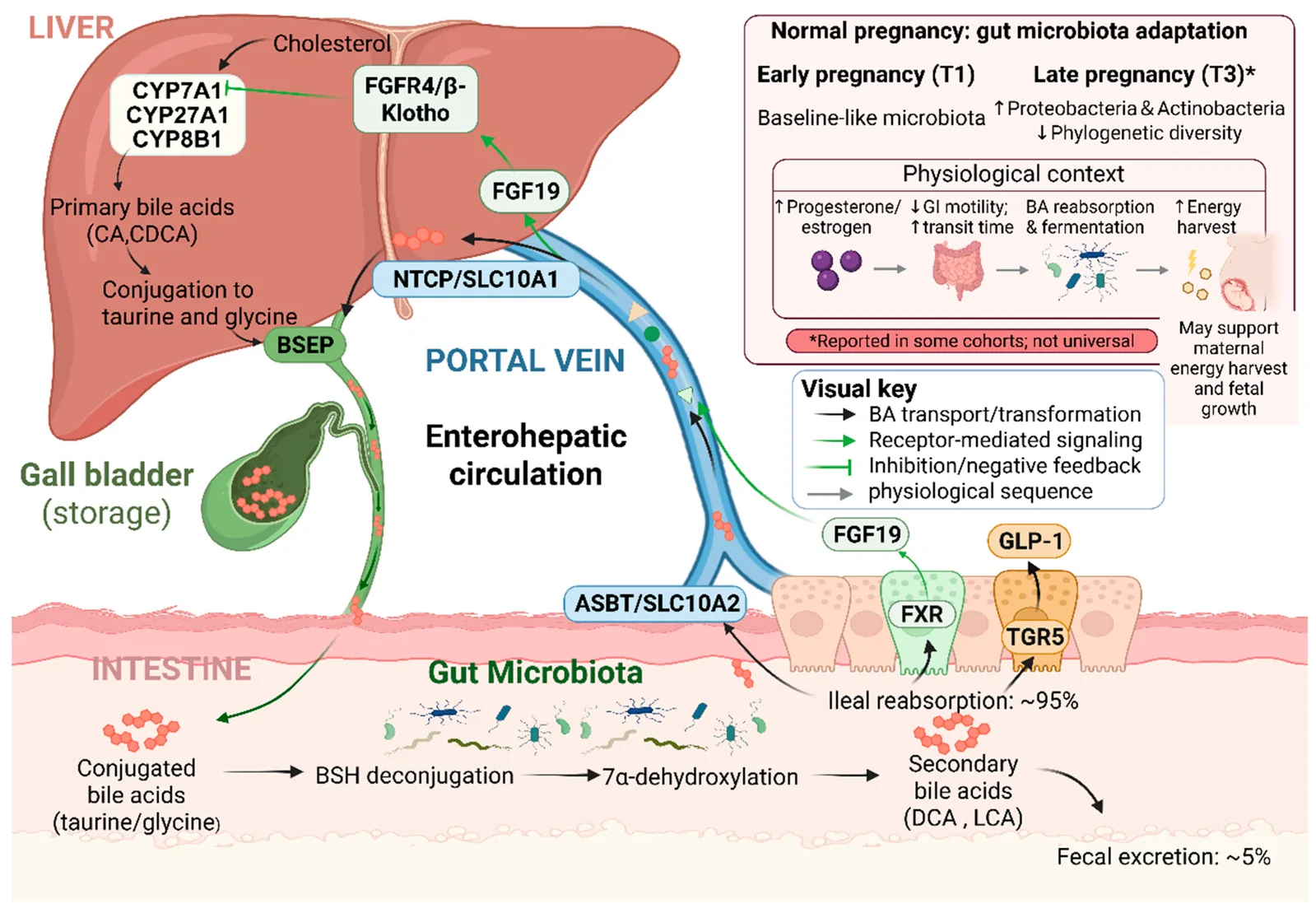
Physiological enterohepatic circulation of bile acids and gut microbiota remodeling during normal pregnancy. Primary bile acids synthesized in the liver are conjugated, secreted into bile, and transformed through BSH-mediated deconjugation and 7α-dehydroxylation. Approximately 95% of intestinal bile acids are reabsorbed and returned to the liver. Ileal FXR–FGF19 signaling suppresses hepatic bile acid synthesis, whereas TGR5 activation promotes GLP-1 release. Pregnancy-related changes in gastrointestinal motility and gut microbial composition may influence bile acid metabolism, although the magnitude and consistency of these changes remain uncertain. Black arrows indicate transport, transformation, or process flow; green arrows indicate receptor-mediated signaling; blunt-ended green lines indicate inhibition; and gray arrows indicate physiological sequence. Upward and downward arrows indicate increases and decreases, respectively. Colors and rounded boxes are used for visual organization only and do not denote additional biological or evidence-level distinctions. Abbreviations: ASBT, apical sodium-dependent bile acid transporter; BA, bile acid; BSEP, bile salt export pump; BSH, bile salt hydrolase; CA, cholic acid; CDCA, chenodeoxycholic acid; *CYP7A1*, cholesterol 7α-hydroxylase; CYP27A1, sterol 27-hydroxylase; CYP8B1, sterol 12α-hydroxylase; DCA, deoxycholic acid; FGF19, fibroblast growth factor 19; FGFR4, fibroblast growth factor receptor 4; FXR, farnesoid X receptor; GI, gastrointestinal; GLP-1, glucagon-like peptide-1; LCA, lithocholic acid; NTCP, sodium taurocholate cotransporting polypeptide; SLC10A1, solute carrier family 10 member 1; SLC10A2, solute carrier family 10 member 2; T1, first trimester; T3, third trimester; TGR5, Takeda G protein-coupled receptor 5.

**Figure 2:**
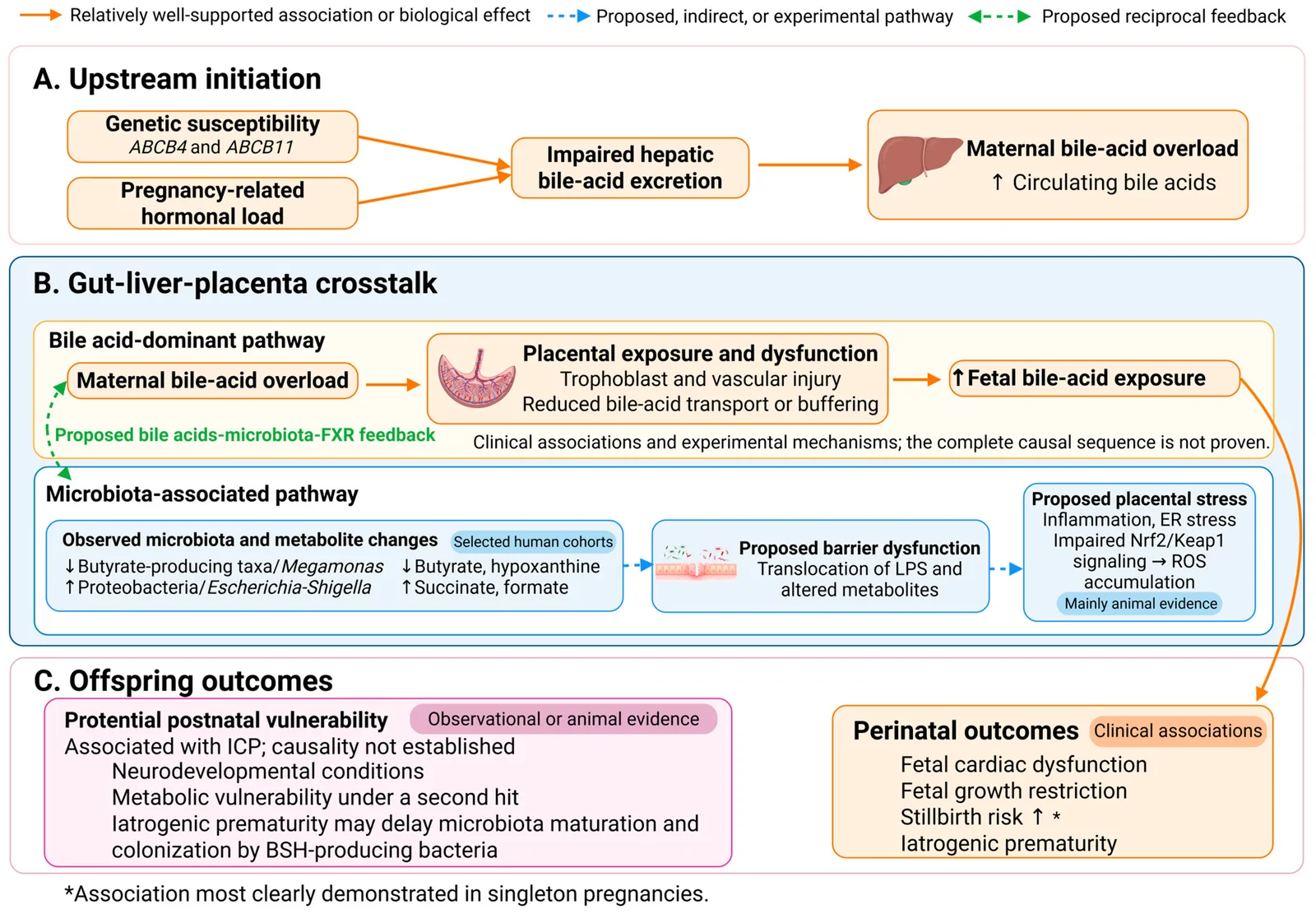
Proposed framework of gut–liver–placenta axis dysregulation and offspring outcomes in intrahepatic cholestasis of pregnancy. (**A**) Genetic susceptibility and pregnancy-related hormonal load contribute to impaired hepatic bile acid excretion, resulting in maternal bile acid accumulation. (**B**) Maternal bile acid overload may promote placental dysfunction and increase fetal exposure. Microbiota and metabolite changes have been observed in selected human cohorts, whereas the proposed downstream effects involving barrier dysfunction, translocation of gut-derived products, and placental Nrf2/Keap1–ROS dysregulation are supported mainly by indirect or animal evidence. (**C**) Perinatal outcomes are clinically associated with ICP, whereas evidence for potential postnatal effects remains primarily observational or experimental. The association between TBA ≥ 100 μmol/L and stillbirth is most clearly demonstrated in singleton pregnancies. Solid orange arrows indicate relatively well-supported associations or biological effects; dashed blue arrows indicate proposed or experimental pathways; and dashed green double-headed arrows indicate proposed reciprocal feedback. Upward and downward arrows indicate increases and decreases, respectively. The asterisk indicates that the association with stillbirth risk has been most clearly demonstrated in singleton pregnancies. Abbreviations: *ABCB4*, ATP-binding cassette subfamily B member 4; *ABCB11*, ATP-binding cassette subfamily B member 11; BSH, bile salt hydrolase; ER, endoplasmic reticulum; FXR, farnesoid X receptor; ICP, intrahepatic cholestasis of pregnancy; LPS, lipopolysaccharide; Nrf2, nuclear factor erythroid 2-related factor 2; Keap1, Kelch-like ECH-associated protein 1; ROS, reactive oxygen species; TBA, total bile acids.

## Data Availability

No new data were created or analyzed in this study. Data sharing is not applicable to this article.
